# Castleman disease: Experience from a single institution

**DOI:** 10.3892/mi.2023.116

**Published:** 2023-10-06

**Authors:** Sherry S. Abraham, Geetha Narayanan, Sugeeth Mangalapilly Thambi, Jayasudha Arundhathi Vasudevan, Deepa Susan Joy Philip, Prakash N. Purushothaman, Sreejith G. Nair, Rekha Nair

**Affiliations:** 1 Department of Medical Oncology, Regional Cancer Centre, Thiruvananthapuram, Kerala 695011, India; 2 Department of Pathology, Regional Cancer Centre, Thiruvananthapuram, Kerala 695011, India

**Keywords:** Castleman disease, unicentric, multicentric, human immunodeficiency virus-negative

## Abstract

Castleman disease (CD) describes a group of rare heterogeneous lymphoproliferative disorders characterized by enlarged hyperplastic lymph nodes. It is classified into unicentric CD (UCD) and multicentric CD (MCD). The present retrospective study examined the data of 11 patients with CD diagnosed and treated at a tertiary cancer center from 2017 to 2022. The median age of the study group was 41 years (range, 24 to 68 years). There were 8 males and 3 females. In total, 7 patients were diagnosed with UCD and 4 patients with MCD. The hyaline-vascular variant was the most common histology in both UCD and MCD. Among the 7 patients with UCD, 5 patients underwent excision, 1 patient underwent debulking followed by radiotherapy and 1 patient received single agent rituximab. Of the patients with UCD, 6 had a complete response (CR) and 1 patient had a partial response (PR). All 4 patients with MCD received systemic treatment, which included single agent rituximab (2 patients), rituximab, cyclophosphamide, doxorubicin, vincristine and prednisolone (RCHOP) (1 patient) and CHOP (1 patient). Among the patients with MCD, 1 patient attained a CR, 2 patients had a PR and 1 patient succumbed. The 3-year survival rate for the study population was 91%. In summary, CD is a rare disease occurring in immunodeficient patients. UCD is more common and is associated with better outcomes. Surgery is the mainstay of management in UCD whereas MCD requires combination chemotherapy.

## Introduction

Castleman disease (CD) describes a group of rare heterogeneous lymphoproliferative disorders characterized by enlarged hyperplastic lymph nodes. Based on the clinical features and distribution of lymphadenopathy, CD can be classified into unicentric CD (UCD) and multicentric CD (MCD) (1). Histopathologically, CD can be classified into the hyaline-vascular (HV) variant, plasma cell (PC) variant and mixed types. The HV type is more common in patients with UCD, whereas the PC type is more commonly observed in MCD (2). MCD is frequently observed in patients with human immunodeficiency virus (HIV) infection (3). Human herpes virus 8 (HHV-8) infection is observed in all HIV-positive patients and in a few HIV-negative patients with MCD (4). Idiopathic MCD (iMCD) refers to those patients without HHV-8 and HIV infection (5).

Patients with UCD often present with lymphadenopathy and compressive symptoms. MCD is frequently associated with systemic manifestations, including fever, fatigue, edema and weight loss (2). Complete resection of the involved lesion is the gold standard of treatment for UCD (6). MCD, and particularly iMCD, is associated a poor prognosis and systemic treatment is preferred (5). The present study examined the demographic characteristics, clinical presentation, histology, staging, treatment and outcomes of 11 patients diagnosed with CD at the Regional Cancer Centre, Thiruvananthapuram, India.

## Patients and methods

The present study was a retrospective study on 11 patients with CD, diagnosed and treated at Regional Cancer Centre from 2017 to 2022. Data on demographic details including age and sex, clinical presentation, laboratory investigations, radiological findings, histopathological details and treatment received were collected from the medical records. All patients underwent a tissue biopsy for a histopathological diagnosis. They were classified as HV, PC and mixed types based on the morphology and immunohistochemistry. Lymph nodes were fixed in 10% neutral buffered formalin at room temperature for a period of 24 to 48 h. Sections were cut at a thickness of 3-4 µm. Immunohistochemistry was performed using the automated Ventana Benchmark XT system. The sections were incubated with primary antibody (CD138, dilution 1:50, Biocare Medical, LLC; clone M115) at 37˚C for 32 min and secondary antibody (ultraview DAB detection kit, ready to use, Ventana labx; Ventana Medical Systems, Inc.) at 37˚C for 12 min. The sections were counterstained with hematoxylin (MilliporeSigma) and visualized under a light microscope (Olympus Corporation).

Based on the anatomical distribution of CD, patients were divided into UCD and MCD. The UCD group consisted of patients who had histological evidence of CD in a single group of lymph nodes without clinical or radiological evidence of adenopathy elsewhere. Patients with MCD had histological evidence of CD in ≥1 group of lymph nodes and radiological or clinical evidence of adenopathy elsewhere (1). Histologically, CD was classified into the HV type and PC type based on the histological criteria proposed by Keller *et al* (7). Lymph nodes with characteristics intermediate between HV and PC were categorized as mixed type (1). The standard of care for UCD is surgical excision and for multicentric CD, systemic therapy is the mainstay of treatment. The response assessment was performed based on the revised RECIST criteria (version 1.1) (8). Follow-up details were noted from the case file. The study was conducted after obtaining patient consent and the approval of the Human Ethics Committee at Regional Cancer Centre.

## Results

**Figure 1:**
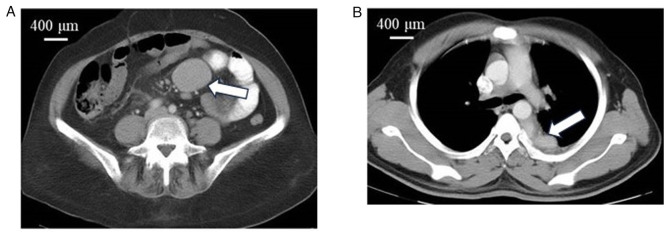
(A) CECT illustrating a mesenteric mass (patient no 4) (arrow). (B) CECT illustrating a posterior mediastinal mass (patient no 6) (arrow).

The median age of the study population was 41 years (range, 24 to 68 years). There were 8 males and 3 females. The baseline clinical characteristics, treatment summary, outcomes and survival are summarized in Table I. Posterior mediastinal mass, colonic mass and jejunal mass, which are rare extranodal presentations, were each observed in 1 patient (patient nos. 6, 7 and 10, respectively) (Fig. 1). The median duration of symptoms was 3 months (range, 1 to 10 months).

**Figure 2:**
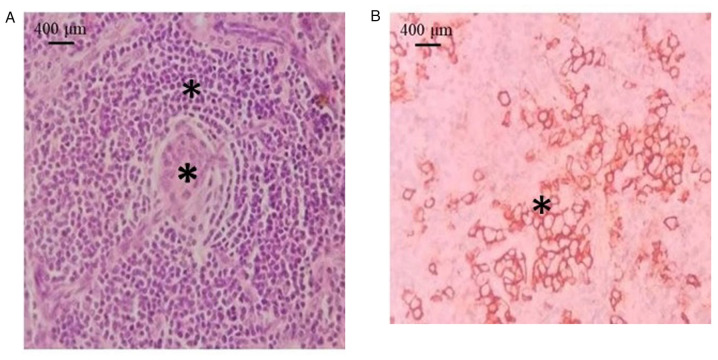
(A) Hyaline-vascular type: Lymphoid follicles can be observed with germinal centers, penetrated by sclerotic blood vessels (lollipop lesions) (indicated by asterisks) and broad mantle zones with concentric rings of small lymphocytes (onion skin pattern) (indicated by asterisks) (hematoxylin and eosin staining; magnification, x400). (B) Mixed type: Interfollicular area is shown with clusters of plasma cells highlighted by CD138 (indicated by asterisks) (hematoxylin and eosin staining; magnification, x400).

A total of 7 patients were diagnosed with UCD and 4 patients were diagnosed with MCD. A comparison of the clinical profiles of the patients with UCD and MCD is presented in Table II. Systemic symptoms, including fever and weight loss were observed in 5 patients, of whom 4 patients had UCD. Histopathologically, the HV variant exhibited small lymphocytes with lymphocyte depletion in germinal centers, penetrated by sclerotic blood vessels (lollipop lesions) and broad mantle zones with concentric rings of small lymphocytes (onion skin pattern) (Fig. 2A). The mixed subtype exhibited clusters of plasma cells in the interfollicular area with cytoplasmic membrane staining for CD138 (Fig. 2B). Among the 7 patients with UCD, 3 patients had the HV subtype, 2 patients had the PC subtype, 1 patient had a mixed histology and in 1 patient, the subtype was not specified. Among the patients with MCD, 2 patients had the HV type and 1 patient each had PC and a mixed histology. In total, 6 patients with UCD had visceral disease presentation. Furthermore, 3 patients with MCD presented with peripheral lymphadenopathy (Table II). In addition, 1 patient (patient no. 11) was positive for HHV-8 and 1 patient (patient no. 2) had associated polyneuropathy, organomegaly, endocrinopathy, monoclonal plasma cell disorder, skin changes (POEMS) syndrome. All patients were negative for HIV (Table II).

Among the 7 patients with UCD, 6 patients underwent surgery, including 5 patients who underwent complete excision, 1 patient with debulking, and 1 patient (patient no. 11) received rituximab. Six patients with UCD attained a complete response (CR) and 1 patient had a partial response (PR). All patients with UCD are alive and on follow-up. The patients with MCD received systemic treatment with rituximab and/or chemotherapy with rituximab, cyclophosphamide, adriamycin, vincristine and prednisolone (R-CHOP). Of the patients with MCD, 1 patient (patient no. 9) attained CR, 2 patients had a PR and are under follow-up. In addition, 1 patient (patient no. 8) succumbed after 3 months. The 3-year survival rate for the study group was 91%.

## Discussion

CD is a rare lymphoproliferative disorder first described by Benjamin Castleman in 1956 in a series of 13 patients with mediastinal mass resembling thymoma (9). In the present study, the authors discuss their experience in treating this rare disease.

Patients with CD usually present in the fourth decade of life (2). The HV variant is commonly observed in UCD and the PC variant is more common in MCD (10). In the study by Yu *et al* (11), the abdomen was the most common site of disease presentation (39.5%) in patients with UCD followed by neck and mediastinum. In the present case series, the median age was 41 years. UCD was the most common type observed in >50% of patients, which is consistent with that reported in previous studies (10,12). In the present study, HV was the most common histology in both UCD and MCD. A total of 6 patients with UCD in the present study had visceral disease and 1 patient had posterior mediastinal mass.

Systemic symptoms and organomegaly are more frequent in patients with MCD (13,14). However, in the present study, systemic symptoms were more common in the UCD type. A total of 2 patients with MCD had hepatosplenomegaly, while none with UCD had the same. The presence of POEMS syndrome is often associated with a significant risk of mortality (14). In the present series, 1 patient (patient no. 2) with POEMS syndrome was alive with CR and on follow-up at 12 years.

Overall survival approaching 100% has been documented for patients with UCD following surgery (15). Talat *et al* (6) studied 278 patients with UCD treated with surgery and reported an overall survivalof 90% at a follow-up of 10 years. In the study by Dispenzieri and Fajgenbaum (16), the 5-year survival rate was 91% for patients with UCD treated surgically. Radiotherapy has a role in patients with unresectable disease or in those who have an incomplete resection (17). In the present study, among the 6 patients with UCD who underwent surgery, 5 patients attained CR and are on follow-up. In addition, 1 patient (patient no. 6) had a PR and is under follow-up with at 3 years.

Systemic therapy is the mainstay of treatment for patients with MCD (13). The monoclonal antibody against anti-CD20, rituximab, has been proven to be effective in HIV-associated MCD. Bower *et al* (18) reported an overall survival rate of 95% at 2 years for patients with HIV-associated MCD treated with rituximab. Rituximab-based therapies have led to a marked improvement in the outcome of these patients with 5-year overall survival rates reaching >90% (19). In patients with iMCD, a 78% overall remission rate has been observed in those treated with chemotherapy; however, many patients subsequently progress or develop infectious complications (20). In the present study, among the 4 patients with MCD who received systemic treatment, 2 patients progressed.

In conclusion, CD is a rare disease occurring in immunodeficient patients. UCD is more common and is associated with improved outcomes. Surgery is the mainstay of management in unicentric disease, whereas MCD requires combination chemotherapy.

## Figures and Tables

**Table I:** Clinical characteristics, treatment summary, outcomes and survival of the study population.

Patient no.	Age, years/sex	Clinical presentation	Stage	Histology	Treatment	Status	Overall survival (months)
1	34/F	Retroperitoneal mass	Unicentric	Hyaline vascular	Nephrectomy and en bloc excision	Alive, complete response	48
2	24/M	Mesenteric mass POEMS syndrome	Unicentric	Mixed	Excision	Alive, complete response	149
3	32/M	Retroperitoneal mass	Multicentric	Hyaline vascular	Rituximab	Alive, partial response	48
4	54/F	Mesenteric mass	Unicentric	Plasma cell	Excision	Alive, complete response	48
5	63/M	Generalised lymphadenopathy	Multicentric	Mixed	Rituximab	Alive, partial response	53
6	25/M	Posterior mediastinal mass	Unicentric	Hyaline vascular	Debulking, radiotherapy	Alive, partial response	40
7	60/M	Colonic mass	Unicentric	Not known	Right hemicolectomy	Alive, complete response	60
8	45/M	Generalized lymphadenopathy	Multicentric	Plasma cell	CHOP 1 cycle	Expired	3
9	25/M	Generalized lymphadenopathy	Multicentric	Hyaline vascular	RCHOP ^*^6	Alive, complete response	14
10	68/F	Jejunal mass	Unicentric	Hyaline vascular	Bowel resection	Alive, complete response	20
11	41/M	External iliac and inguinal adenopathy	Unicentric	Plasma cell	Rituximab	Alive, complete response	10

M, male; F, female; POEMS, polyneuropathy, organomegaly, endocrinopathy, monoclonal plasma cell disorder, skin changes.

**Table II:** Comparison of the clinical profiles of patients with unicentric and multicentric Castleman disease.

Characteristic	Unicentric (n=7)	Multicentric (n=4)
Age (years), median (range)	41 (24-68)	38.5 (25-63)
Sex		
Male	4	4
Female	3	-
Location		
Peripheral	1	3
Visceral	6	1
Systemic symptoms	4	1
Lymphadenopathy	1	3
Hepatosplenomegaly	-	2
Anemia	2	2
Hypoalbuminemia	2	2
Pathological subtype		
Hyaline-vascular	3	2
Plasma cell	2	1
Mixed	1	1
Human herpes virus 8	1	-
POEMS syndrome	1	-

POEMS, polyneuropathy, organomegaly, endocrinopathy, monoclonal plasma cell disorder, skin changes.

## Data Availability

The datasets used in the current study are available from the corresponding author upon reasonable request.
